# Genome-wide SNP scan of pooled DNA reveals nonsense mutation in *FGF20* in the scaleless line of featherless chickens

**DOI:** 10.1186/1471-2164-13-257

**Published:** 2012-06-19

**Authors:** Kirsty L Wells, Yair Hadad, Danny Ben-Avraham, Jossi Hillel, Avigdor Cahaner, Denis J Headon

**Affiliations:** 1 The Roslin Institute and Royal (Dick) School of Veterinary Studies, University of Edinburgh, Midlothian, EH25 9RG, United Kingdom; 2 Faculty of Agriculture, Food and Environment, The Hebrew University of Jerusalem, P.O Box 12, Rehovot, 76100, Israel

## Abstract

**Background:**

Scaleless (*sc/sc*) chickens carry a single recessive mutation that causes a lack of almost all body feathers, as well as foot scales and spurs, due to a failure of skin patterning during embryogenesis. This spontaneous mutant line, first described in the 1950s, has been used extensively to explore the tissue interactions involved in ectodermal appendage formation in embryonic skin. Moreover, the trait is potentially useful in tropical agriculture due to the ability of featherless chickens to tolerate heat, which is at present a major constraint to efficient poultry meat production in hot climates. In the interests of enhancing our understanding of feather placode development, and to provide the poultry industry with a strategy to breed heat-tolerant meat-type chickens (broilers), we mapped and identified the *sc* mutation.

**Results:**

Through a cost-effective and labour-efficient SNP array mapping approach using DNA from *sc/sc* and *sc/+* blood sample pools, we map the *sc* trait to chromosome 4 and show that a nonsense mutation in *FGF20* is completely associated with the *sc/sc* phenotype. This mutation, common to all *sc/sc* individuals and absent from wild type, is predicted to lead to loss of a highly conserved region of the FGF20 protein important for FGF signalling. *In situ* hybridisation and quantitative RT-PCR studies reveal that *FGF20* is epidermally expressed during the early stages of feather placode patterning. In addition, we describe a dCAPS genotyping assay based on the mutation, developed to facilitate discrimination between wild type and *sc* alleles.

**Conclusions:**

This work represents the first loss of function genetic evidence supporting a role for FGF ligand signalling in feather development, and suggests FGF20 as a novel central player in the development of vertebrate skin appendages, including hair follicles and exocrine glands. In addition, this is to our knowledge the first report describing the use of the chicken SNP array to map genes based on genotyping of DNA samples from pooled whole blood. The identification of the *sc* mutation has important implications for the future breeding of this potentially useful trait for the poultry industry, and our genotyping assay can facilitate its rapid introgression into production lines.

## Background

Avian skin carries feathers and scales which commence development prior to hatching. In birds, feathers develop within specific regions of the skin called tracts, while scales and spurs form only on the legs and feet [1]. Before the development of cutaneous appendages, the embryonic skin consists of two morphologically homogenous tissue layers: the dermis and the overlying epidermis. Classical tissue recombination experiments have shown that the development of feather and scale primordia is achieved through communication between these two tissue layers [2,3]. Prior to the morphological appearance of feather rudiments, the dermal cells proliferate to form a dense tissue layer. Signalling from the dense dermis induces rows of overlying epidermal cells to become competent to form the feather primordia. These rows, which can be visualised with molecular markers, break up to form distinct, circular epidermal condensations called placodes [4-6]. This process occurs in a wave which travels laterally across the skin, laying down the feather pattern row by row. Signalling from the patterned epidermis induces separate dermal condensations to form underneath each placode, and individual placodes undergo rapid proliferation to form outgrowths. Subsequent branching and differentiation result in the formation of a mature feather [6].

The early stages of chicken skin patterning involve a number of activatory and inhibitory molecular signals. WNT/β-catenin signalling plays an activatory role, since forced activation of this pathway results in the formation of ectopic feather buds in embryonic chicken skin [4,7]. FGF signalling also appears to be activatory as overexpression of dominant negative FGF receptors in embryonic chicken skin suppresses feather placode formation [8], while application of recombinant FGFs can induce placodes [9]. Members of the BMP family are considered to act as opposing inhibitory factors; retroviral expression of BMP2 or 4 in chicken skin, or application of recombinant BMP protein to skin in culture, blocks feather placode formation [5,10,11]. These experiments have begun to reveal the key pathways involved in chicken skin patterning, but genetic evidence implicating the specific molecules acting in these multi-component pathways is as yet lacking.

**Figure 1:**
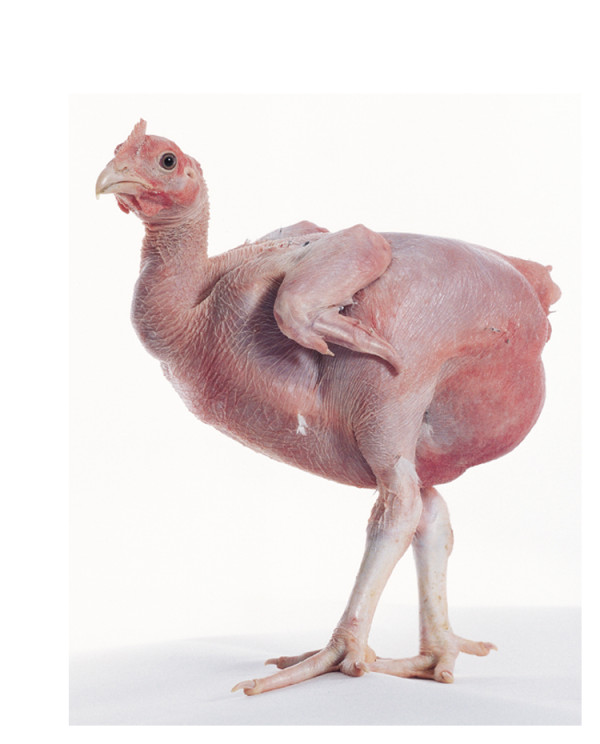
**The scaleless phenotype.** Gross appearance of a *sc/sc* chicken. The majority of feathers and all scales are absent.

Mutants in which feather development is affected offer a valuable opportunity to explore the developmental mechanisms involved in ectodermal appendage formation from embryonic skin. Scaleless (*sc/sc*) is a chicken mutant which lacks almost all feathers, as well as scutate scales and spurs [12]. The defect is known to originate in embryogenesis when feather and scale placodes fail to form [4,13]. This dramatic trait (Figure 1) is inherited in an autosomal recessive pattern, and has yet to be genetically characterised. The *scaleless* (*sc*) mutation originated in a flock of New Hampshire chickens at the University of California, Davis in 1954 [12]. The original mutant line was subjected to outcrossing and selection for cold tolerance to generate two divergent lines; the scaleless low line which has no scales and is almost completely featherless, exhibiting only very few feathers which typically form on the thigh; and the scaleless high line, which also exhibits no scales but has greater feather coverage than the low line due to unlinked modifier alleles [14]. *sc/sc* mutants have been used extensively since the 1960s to study the tissue interactions which take place during skin development. These studies have shown that reciprocal signalling between the epidermal and dermal tissue layers is required during feather patterning, and that the timing of these signals is key [13,15,16]. The *sc* gene also appears to be redundantly involved in chicken limb development as the *ectrodactyly* mutation, identified in the 1960s, produces limb and beak defects on the scaleless background, but results in beak deformities only on a wild type background [17]. In addition to analyses of embryonic processes, *sc/sc* chickens have been used in a number of behavioural studies, including investigations into the acquired versus innate nature of dustbathing and wing flapping [18,19].

Detailed studies of developing *sc/sc* skin have shown that dense dermis forms in these mutants, but subsequent epidermal placode formation does not occur [13]. Tissue recombination experiments indicated that the primary defect is epidermal, since recombination between wild type epidermis and *sc/sc* dermis results in placode formation, whereas the reverse combination of *sc/sc* epidermis and wild type dermis does not [13]. Molecular markers of feather patterning, such as *CTNNB1* and *EDAR*, are expressed broadly in *sc/sc* embryonic skin, but their expression does not resolve into a punctate pattern [4,20], or is only transiently punctate in some regions of the body [21], suggesting that the epidermis is unable to participate in pattern formation. Dermal condensations do not form in *sc/sc* skin and the dermal condensate marker *Delta-1* remains diffusely expressed throughout the dermis [22]. Thus the molecular identification of the *sc* gene will define a key endogenous regulator of feather patterning.

*sc/sc* mutants are also potentially useful for meat production in hot climates. Hot conditions depress the growth rate of modern meat-type chickens (broilers) due to elevation in body temperature, and consequently they yield less meat with poorer feed efficiency [23]. Therefore, in hot climates broiler houses require costly and polluting cooling systems. Reduced feather coverage improves heat tolerance [24-27]. As a result, featherless broilers do not suffer in hot conditions; they grow normally, yield significantly more breast meat and display better viability than feathered broilers [28-31]. Thus breeding for reduction or elimination of feather coverage offers a cost effective, animal-friendly and environmentally-friendly approach to broiler meat production in hot climates.

To enhance our understanding of feather development, and to facilitate efficient introgression of the featherless trait into production lines, we sought to identify the *sc* mutation. We mapped *sc* to a region on chromosome 4 and show that a nonsense mutation in *FGF20*, a gene expressed in developing feathers, is completely associated with the *sc/sc* phenotype.

## Results

**Figure 2:**
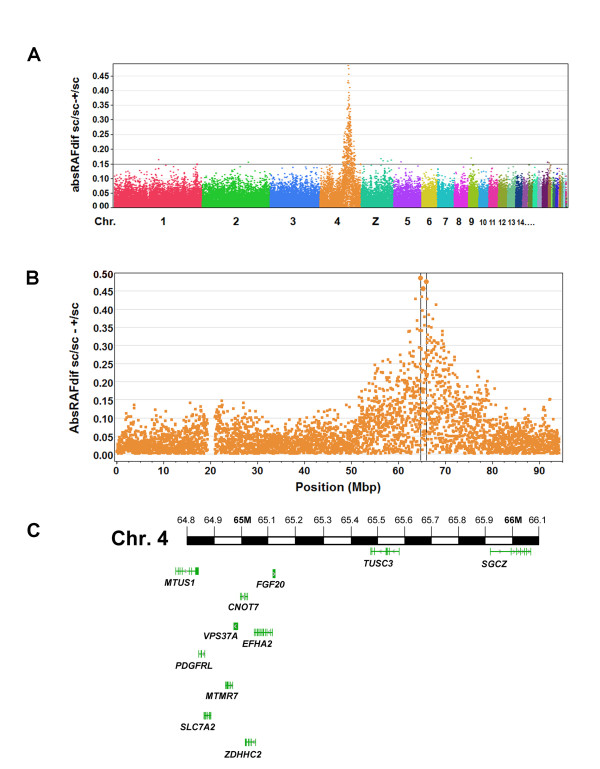
**Mapping of the*scaleless*mutation.** (**A**) Genome-wide absRAFdif values (calculated by contrasting *sc/+* and *sc/sc* RAF values) of all 60 K SNPs in the GWMAS consortium chicken SNP array, plotted against genomic location. A peak of values is apparent on chromosome 4. (**B**) absRAFdif values on chromosome 4. Three SNPs located within a 1.25 Mb region (boxed area) gave absRAFdif values above 0.45, suggesting the location of the *sc* mutation. (**C**) Schematic of genes present within the 1.25 Mb region defined by mapping.

We used the Illumina 60 K chicken SNP chip to map the *sc* locus based on genotyping of DNA from pooled samples of blood [32]. Blood pools were prepared from 86 *sc/sc* individuals and from 120 *sc/+* individuals, which were segregating progeny of the same *sc/sc* sires and *sc/+* dams and thus shared a similar genetic background. DNA extracted from each of these two pools was hybridised to the chip. The intensity readings at each of the SNP’s alleles were used to calculate relative allelic frequency (RAF). The absolute difference in RAF (absRAFdif) between the *sc/sc* and *sc/+* groups was plotted against the genomic location of each SNP. One hundred and fifty seven SNPs, all located within an 18 Mb region of chromosome 4 (Figure 2A), showed exceptionally high absRAFdif values. Of these, a peak of 3 SNPs all with an absRAFdif value of >0.45, was located within a 1.25 Mb region (4:64,773,116 – 4:66,022,973), suggesting this region on chromosome 4 as the location of the *sc* mutation (Figure 2B).

To identify the *sc* mutation, we amplified and sequenced the exons of all 11 genes within the 1.25 Mb mapped region (Figure 2C). The coding sequences of all candidate genes were amplified from *sc/sc* cDNA or genomic DNA (gDNA) and compared to the chicken reference genome. We found 1 previously unknown SNP within an *EFHA2* predicted splice site, 2 SNPs within *SLC7A2* predicted to lead to nonsynonymous changes, and a SNP in exon 3 of *FGF20* predicted to result in a premature stop codon. We sequenced these changes from the gDNA of WT chickens from diverse breeds and found all changes associated with *EFHA2* and *SLC7A2* to be carried by most WTs sampled (Additional file 1: Table S1), indicating that they are neutral polymorphisms and do not cause the *sc/sc* phenotype.

**Figure 3:**
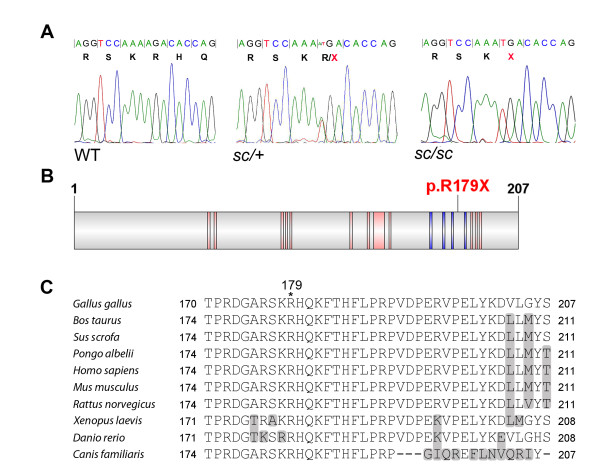
**Identification of a nonsense mutation in*FGF20*in scaleless.** (**A**) Sequence traces from WT, *sc/+* and *sc/sc* individuals covering c.526 to c.543 of *FGF20,* showing the open reading frame, c.535A > T mutation in *sc/sc* and corresponding p.R179X in the predicted protein sequence. (**B**) Schematic of the chicken FGF20 protein. Pink bars indicate predicted receptor interaction sites and blue bars indicate HSPG binding motifs (information obtained from NCBI Domains, accessed 17/8/11: http://www.ncbi.nlm.nih.gov/Structure/cdd/wrpsb.cgi). The p.R179X mutation is predicted to cause loss of receptor and HSPG binding sites. (**C**) Multiple sequence alignment of vertebrate FGF20 sequences to amino acids 170 to 207 of chicken FGF20. The position of the premature termination caused by the *scaleless* mutation in chicken FGF20 is indicated above the alignment. Sequence divergence is illustrated by shading of amino acids. The residues downstream of chicken FGF20 R179 are highly conserved across species.

We sequenced exon 3 of *FGF20* in 40 WT birds of diverse breeds and found that the nonsense mutation (c.535A > T) (Figure 3A) was absent in all of the samples sequenced (Table 1). We then sequenced exon 3 of *FGF20* in 38 *sc/sc* individuals and 9 *sc/+* individuals from different sources. All of the *sc/sc* samples sequenced were homozygous for c.535A > T and all of the *sc/+* individuals were heterozygous for the mutation (Table 1), supporting the idea that *FGF20* c.535A > T is the *sc* mutation. The c.535A > T mutation is predicted to lead to the production of a truncated protein lacking 29 amino acids from the C-terminus of FGF20. The truncated region is highly conserved across species and encodes motifs associated with receptor interaction and binding of heparan sulphate proteoglycans (HSPG) (Figure 3B,C).

**Table 1:** **Breed and genotype of individuals sequenced for the presence of*FGF20*c.535A > T**

**Breed**	**Genotype**	**No. of individuals**	**c.496 (rs14481412)**	**c.535**	**c.552 (rs14481413)**
UC Davis (low line)	*sc/sc*	10	CC	TT	AA
UC Davis (high line)	*sc/sc*	10	CC	TT	AA
Storrs, Connecticut (low line)	*sc/sc*	2	CC	TT	AA
Israeli experimental line	*sc/sc*	16	CC	TT	AA
Israeli experimental line	*sc/+*	3	CC	AT	AA
Israeli experimental line	*sc/+*	6	CC	AT	AG
New Hampshire (IFAG, Germany)	WT	6	CC	AA	AA
New Hampshire (IFAG, Germany)	WT	4	CT	AA	AG
New Hampshire, UK	WT	2	TT	AA	GG
New Hampshire, UK	WT	1	CT	AA	AG
Black Leghorn	WT	1	CC	AA	AA
Araucana	WT	1	CC	AA	AA
Old English Pheasant Fowl	WT	1	CC	AA	AA
Langshan	WT	1	CT	AA	AG
Silver Appenzeller	WT	1	CC	AA	AA
Roslin Institute experimental	WT	2	CC	AA	AA
Roslin Institute experimental	WT	1	CT	AA	AG
ISA Brown	WT	2	CC	AA	AA
Outbred, Mexico	WT	7	CC	AA	AA
Outbred, Mexico	WT	2	TT	AA	GG
Outbred, Mexico	WT	1	CT	AA	AG
Cream Legbar	WT	1	CC	AA	AA
Dorking	WT	1	CC	AA	AA
Campine	WT	1	CC	AA	AA
Naked Neck, Wernlas collection	WT	1	TT	AA	GG
Hungarian AVIANDIV Naked Neck	WT	1	TT	AA	GG
Naked Neck, English	WT	1	CC	AA	AA
Scots Grey	WT	1	TT	AA	GG

Results from sequencing *FGF20* exon 3 in 38 *sc/sc* individuals, 9 *sc/+* individuals and 40 WT birds. The *FGF20* c.535A > T mutation was found in all of the *sc/sc* and none of the WT samples sequenced. The genotype of the two flanking SNPs (rs14481412 and rs14481413) revealed that 25/40 WT individuals were homozygous for the same haplotype as scaleless, indicating that the *FGF20* c.535A > T mutation does not lie within a rare haplotype.

To confirm that c.535A > T is not a neutral polymorphism present in the WT population we developed a dCAPS genotyping assay to discriminate between WT and *sc* alleles. Using our dCAPS assay it was possible to discriminate WT, *sc/+* and *sc/sc* individuals (Additional file 2: Figure S1). DNA from 92 wild type chickens from diverse traditional breeds did not carry the c.535A > T mutation (Table 2). The absence of this mutation from 264 WT chromosomes strongly indicates that it is not a neutral polymorphism but is the causative mutation for the scaleless phenotype.

**Table 2:** **WT individuals assayed by dCAPS for presence of*FGF20*c.535A > T**

**Breed**	**No. of individuals**
Appenzeller	4
Araucana	4
Brahma	3
Buff Orpington	4
Cochin	3
Croad Langshan	4
Derbyshire Redcap	4
Dorking	4
Hamburg	4
Indian Game	4
Ixworth	4
Leghorn (coloured)	4
Lincolnshire Buff	4
Maran	4
Marsh Daisy	4
Norfolk Grey	4
Old English Pheasant Fowl	4
Rhode Island Red	3
Scots Dumpy	4
Scots Grey	3
Silkie	4
Spanish	4
Light Sussex	4
Sussex	4

DNA was obtained from 24 breeds of chicken [33] consisting of individuals from 3 or 4 flocks to maximise between- and within-breed genetic diversity.

**Figure 4:**
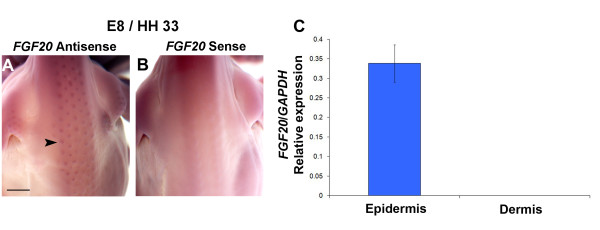
**Expression of*FGF20*in developing feather placodes.** (**A,B**) E8 (Hamburger-Hamilton stage 33) WT chicken embryos (dorsal view) hybridised with a digoxigenin-labelled riboprobe specific for *FGF20* (antisense) (**A**) or a negative control *FGF20* riboprobe (sense) (**B**). Punctate purple staining indicates specific expression of *FGF20* in developing feather placodes (arrowhead). Scale bar = 500 μm. (**C**) Quantitative RT-PCR assessing *FGF20* expression levels in separated dermis and epidermis from E8 WT chicken skin. Expression is specific to the epidermis. Error bars indicate SEM.

We sought to define the expression pattern of *FGF20* to confirm its role in feather development. As the *sc/sc* phenotype is apparent by the 8^th^ day of *in ovo* development, when feather placodes form in WT embryos but fail to appear in the mutant, the mutated gene must be expressed by this developmental stage. Therefore, we performed *in situ* hybridisation to embryonic day (E) 8 WT chicken embryos using a riboprobe specific for *FGF20*. Consistent with a role for *FGF20* in early feather development, we found that it is expressed in the developing feather placodes (Figure 4A,B). In addition, using qRT-PCR we found that *FGF20* is expressed exclusively in the epithelial (ectodermal) component of the skin (Figure 4C), agreeing with tissue recombination experiments which demonstrated that the gene mutated in *sc* must be active in the epidermis [13].

## Discussion

Here we report the identification of a nonsense mutation in *FGF20* in the scaleless chicken line. To identify the *sc* mutation, we performed a genome-wide SNP scan of DNA from two pools of blood sampled from individuals homozygous for the mutation, and from heterozygous individuals. The availability of genome sequences has meant that Genome- Wide Association Studies (GWAS) using dense SNP arrays have become the method of choice when attempting to map loci associated with specific traits. However, cost presents a major hurdle in the effort to genotype the large number of individuals needed for a reliable GWAS. In the early 1990s, several studies demonstrated the use of blood or DNA pools in poultry DNA profiling [34-36]. More recently, a number of human genetics studies have validated the cost-effectiveness, labour-efficiency and reliability of using DNA pools in GWAS [37-39]. Our results demonstrate that, using a 60 K chicken SNP array and DNA from two contrasting blood sample pools, a single mutation underlying a qualitative trait can be mapped to within approximately 1.25 Mb. To our knowledge, this is the first report outside the human genetics field describing the use of a SNP array to map genes based on genotyping of DNA from just two whole blood sample pools.

The FGF gene family comprises 22 members encoding secreted signalling molecules with a high affinity for HSPG and for specific members of the FGF receptor family [40]. Compared to other FGF family ligands, FGF20 has a broad receptor binding specificity, and can signal via certain splice variants of all 4 FGF receptors [41,42]. *FGF20* has been implicated in diverse developmental and functional processes, though its roles in vertebrate biology have only recently begun to be discovered. Early studies indicated an important role for FGF20 in development and tumourigenesis, based on its expression profile and mode of regulation [41,43]. More recently, it has been reported that an apparent gain of function allele of *FGF20* is associated with Parkinson's disease and brain structure in human populations [44-46]. In the zebrafish, fin regeneration after injury has been shown to require a functional *fgf20a* gene, one of the two orthologues of amniote *FGF20* in the fish genome [47]. The phenotype of *Fgf20* null mice remains to be fully described, however it is reported that development of the organ of corti and thus normal hearing is disrupted in this model [48]. The work we report here represents the first evidence supporting a role for *FGF20* in integumentary appendage development. Given the similarities between the early stages of feather and hair development [49], *FGF20* may be involved in mammalian hair placode patterning. Assessment of this idea requires detailed analyses of hair follicle development in *Fgf20* null mice, as different hair types in the mouse coat rely on distinct signals for their formation, and often mutant animals display loss or alteration of only one hair type [49].

Our findings are in agreement with previous work implicating FGF signalling in feather placode induction. Overexpression of dominant negative FGF receptors in embryonic chicken skin explants blocks feather formation [8], while application of recombinant FGF1, 2 or 4 can induce placode formation, even in regions of the skin where placodes do not normally form [9]. A study in 1996 demonstrated the ability of recombinant FGF2 to rescue feather placode patterning in *ex ovo* cultured *sc/sc* embryonic skin [50]. It is likely that the overlapping receptor binding specificities of FGF2 and FGF20, particularly their shared ability to stimulate the mesenchymally expressed c-spliced isoforms of FGF receptors [42], allow this partial rescue of *sc/sc* skin. This *ex ovo* rescue study [50] implicated defective FGF signalling as underlying the failure of feather development in *sc/sc*, though such mutant rescue data should be interpreted cautiously in the absence of genetic evidence, since it is possible that such phenotypic rescues are achieved via a pathway independent or downstream of the primary causative genetic defect. For example, it has been shown that tooth and gland defects in *Eda* mutant mice can be rescued with recombinant Fgf10 and Shh, as well as by recombinant Eda itself [51,52].

This work represents the first genetic evidence supporting a role for FGF signalling in feather development, and complements previous work identifying roles for FGF signalling in the development of mouse [53,54] and fish [55] ectodermal appendages. Our evidence is strengthened by our experiments showing expression of *FGF20* at the expected developmental stage and in the appropriate tissue layer. In addition, our dCAPS test can be used to speed the introgression of the *sc* mutation into contemporary production lines, by facilitating discrimination between the *sc/+* and WT individuals amongst the all-feathered progeny of *sc/+* sires mated to WT dams within a backcross breeding scheme.

## Conclusions

We have shown that scaleless chickens carry a nonsense mutation in *FGF20* and that this gene is epidermally expressed at an early stage in feather placode development. To our knowledge, this is the first report describing the use of the chicken SNP array to map genes based on genotyping of DNA from pooled samples of blood. In addition, this work represents the first loss of function genetic evidence supporting a role for FGF signalling in feather development, and identifies FGF20 as a previously unrecognised central player. The identification of the *sc* mutation also uncovers a role for chicken FGF20 in the development of scales, spurs and limbs. Our work has important implications for the future breeding of this potentially useful line for the poultry industry, and our genotyping assay can facilitate the rapid introgression of this trait into production lines due to its ability to identify *sc* carriers (*sc/+*) in a breeding programme.

## Methods

### Mapping of *sc*

To map the *sc* mutation we used the 60 K SNP Illumina iSelect chicken array developed by USDA Chicken GWMAS Consortium, Cobb Vantress, and Hendrix Genetics. DNA extracted from *sc/sc* and *sc/+* blood pools was genotyped using the Illumina Infinium genotyping system. This system generates an intensity reading for the 2 alleles (X and Y) at each of the 56,702 SNPs in the array. The relative allelic frequencies (RAF) at each SNP for each group were calculated as the ratio of X/(X + Y), where X and Y represent the intensity signals at the SNP's two alleles. For each SNP, absolute RAF difference (absRAFdif) was calculated between the RAF of the *sc/sc* group and the RAF of the *sc/+* group. The absRAFdif is expected to be 0.5 for an informative SNP fully linked to the *sc* mutation and 0 for an unlinked SNP (Additional file 1: Table S2). To map the segregating SNPs, absRAFdif values were plotted against the SNP genomic locations.

### PCR, sequencing and sequence analysis of *sc* candidate genes

*sc* candidate genes were amplified by PCR from cDNA or gDNA derived from a day 4 *sc/sc* embryo, or from adult *sc/sc* gDNA. cDNA was synthesised from 1 μg of RNA extracted using the RNeasy kit (Qiagen). gDNA was extracted by phenol chloroform extraction and precipitation with sodium acetate and ethanol. The coding sequences of candidate genes were amplified from cDNA as far as possible by placing oligonucleotides within predicted 5’ and 3’ UTRs. Regions that failed to amplify from cDNA were amplified exon-by-exon from gDNA using oligonucleotides placed within introns flanking the target exon. Coding sequences were as given by NCBI, except in a few cases in which the NCBI gene model was judged to be incorrect based on EST evidence. Potentially functional mutations were defined as nonsynonymous coding sequence alterations, or any sequence alterations within 10 bases of an intron/exon boundary. All potentially functional mutations were assessed for their presence in databases of known SNPs (ENSEMBL and dbSNP) and sequence changes detected in *sc/sc* which were absent from these databases were then sequenced from WT individuals.

PCRs were performed in a 30 μl reaction volume containing 0.5 μl of FastStart Taq polymerase (Roche), 0.6 μl of 10 mM dNTPs (Roche) and 1.5 μl of a 10 μM dilution of each oligonucleotide. Typical cycling conditions were 96°C 2 min 30 s, 94°C 30 s, 58°C 30 s, 72°C 1 min for 38 cycles. Adjustments to the annealing temperature were made to improve oligonucleotide specificity if required. After treatment with Exonuclease I (NEB) and Shrimp Alkaline Phosphatase (GE Healthcare), PCR products were sequenced with the primers used for PCR amplification, using reagents from the BigDye Terminator v3.1 Cycle Sequencing Kit (Applied Biosystems). Cycle sequencing conditions were: 96°C 3 min, 96°C 30 s, 50°C 20 s, 60°C 4 min, for 25 cycles. Sequences were determined in an ABI 3730xl capillary sequencer.

Oligonucleotides FGF20_9F and FGF20_9R (see supplementary material) were used for PCR and sequencing of the mutated region in *FGF20* exon 3. GC-rich reagent (Roche) was added to the PCR mix. Cycling conditions were 96°C 4 min, 96°C 1 min, 58°C 30 s, 72°C 3 min for 38 cycles.

### dCAPS genotyping

*sc/sc*, *sc/+* and WT DNA samples were amplified from 10 – 500 ng of template DNA in a 15 μl reaction volume containing 0.5 μl of Taq polymerase, 0.5 μl of 10 mM dNTPs (Roche) and 0.5 μl of a 10 μM dilution of each oligonucleotide. Oligonucleotide sequences were: Sc_dCAPS_F:5’-CACTTAACAAAGATGGTACTCCCAGAGATGGAGCAAGGTCCACA-3’ and Sc_dCAPS_R:5’-AAATCTGTCCATCACGAAGTAG-3’. PCR cycling conditions of 96°C 2 min, 94°C 30 s, 55°C 30 s, 72°C 1 min for 36 cycles were used. PCR products were digested overnight at 37°C in a 20 μl reaction volume containing 15 μl of PCR product, 2 μl of NEBuffer 4 and 10 units of NlaIII (NEB). Bands were visualised on a 3% agarose gel containing SYBR® Safe DNA stain (Invitrogen).

### Chicken DNA samples

The WT chicken DNA collection used for dCAPS screening was provided by Dr P. M. Hocking, Roslin Institute, UK and is described in [33]. New Hampshire DNA was obtained in 2012 from a UK based breeder and, separately, from the Institute of Farm Animal Genetics, Friedrich Loeffler Institut, Germany. *sc/sc* and *sc/+* DNA was obtained from 3 separate populations maintained at Storrs, Connecticut, USA; UC Davis, California, USA, and the Hebrew University of Jerusalem, Israel. DNA samples from *sc/sc* low line and high line individuals were obtained from UC Davis in 2012. The *sc/sc* individuals from the University of Connecticut at Storrs were of the scaleless low line and were obtained in 2003 and 2004. The Israeli experimental birds were derived from *sc/sc* individuals obtained from Storrs, Connecticut in 2000, and repeatedly crossed to several fast-growing commercial broiler stocks for 10 generations.

### *In situ* hybridisation

White Leghorn embryos were fixed overnight in 4% paraformaldehyde (PFA) in PBS at 4 °C. Samples were dehydrated into methanol, bleached in 6% H_2_O_2_, rehydrated and treated with 5 μg/ml proteinase K for 3.5 minutes at room temperature. After post-fixing in 4% PFA for 20 minutes, embryos were hybridised with probe at 60°C overnight in 50% formamide, 5 X SSC, 1% SDS, 50 μg/ml heparin and 50 μg/ml yeast RNA in DEPC-treated H_2_O. Samples were washed to remove unbound probe and signal detected using an alkaline phosphatase conjugated sheep anti-digoxigenin antibody (Roche, 1:2000 dilution) and a BCIP/NBT colour reaction. Riboprobes were transcribed from a chicken EST clone (ChEST264g5; GenBank accession: Bu472200.1), contained entirely within the 3’ UTR of *FGF20.*

### Dermis/epidermis separation and quantitative RT-PCR

Dorsal skin of White Leghorn embryos was dissected and incubated in 2 mg/ml Dispase in PBS for 5 minutes at 37°C. Total RNA was isolated from each tissue compartment using TRIZOL (Invitrogen). cDNA was synthesised from 1 μg of total RNA using random primers and AMV reverse transcriptase (Roche) in a 10 μl total volume. Reactions were diluted 10-fold and 3 μl was used as template for each quantitative RT-PCR. Each reaction was performed in a 20 μl total volume using Universal SYBR Green Master Mix (Roche) which contains Rox reference dye. Reactions were performed in triplicate, and four biological replicates were used to determine each data point. *FGF20* expression levels were normalised to those of *GAPDH*. Expression levels for *FGF20* and *GAPDH* were determined from cDNA dilution standard curves. Primer sequences were: GAPDH_270711_F:5’-GACAACTTTGGCATTGTGGA-3’, GAPDH_270711_R:5’-GGCTGTGATGGCATGGAC-3’, FGF20_270711_F1:5’-GCCAAGACCACAGCCTCTT-3’, FGF20_270711_R1:5’-TTCCAAGGTAAAGGCCACTG-3’.

Protocols for chicken rearing and collection of blood samples were approved by the Hebrew University Ethics Committee for Animal Care and Use**.**

## Abbreviations

absRAFdif, Absolute difference in RAF values; BMP, Bone Morphogenetic Protein; dCAPS, Derived Cleaved Amplified Polymorphic Sequences; FGF, Fibroblast Growth Factor; GWAS, Genome-Wide Association Study; GWMAS, Genome-Wide Marker-Assisted Selection; HSPG, Heparan Sulphate Proteoglycan; RAF, Relative Allelic Frequency; RT-PCR, Reverse Transcriptase Polymerase Chain Reaction; sc, scaleless; SNP, Single Nucleotide Polymorphism; WT, Wild Type.

## Competing interests

The authors declare that they have no competing interests.

## Authors’ contributions

KLW: Performed experiments, analysed data and wrote the manuscript. YH: Performed experiments, prepared samples and analysed data. DBA: Performed bioinformatics analyses. JH: Oversaw SNP genotyping. AC: Conceived of the study, developed the experimental population and the mapping approach, analysed data and revised the manuscript. DJH: Conceived of the study, performed experiments, analysed data and revised the manuscript. All authors read and approved the final manuscript.

## Supplementary Material

Additional file 1 **Table S1.** Results from sequencing of candidate mutations in*EFHA2*and*SLC7A2.*The polymorphisms identified in *sc/sc* were found to be carried by a number of WTs. The nucleotide numbers given for the *EFHA2* c.144 and *SLC7A2* c.1211 SNPs refer to the NCBI coding sequences; the nucleotide numbers given for the *SLC7A2* polymorphisms between c.1159 and c.1164 refer to the ENSEMBL coding sequence. The letter/number codes given for each wild type sample are our identifiers for specific individuals within the diverse chicken DNA collection. **Table S2** Raw data obtained from mapping of the*sc*locus. Names, position on chromosome 4, intensity values and absRAFdif for each SNP located within the 1.25 Mb mapped region depicted in Figure 2 are shown, together with the location of each of the 11 genes in the region (shaded). The 3 SNPs with absRAFdif values above 0.45 are highlighted in red. See Methods for explanation of data processing. Click here for file

Additional file 2 **Figure S1.**Genotyping assay to discriminate*FGF20* c.535A and*FGF20* c.535T alleles. Agarose gel analysis of undigested dCAPS PCR product, and WT, *sc/+*, and *sc/sc* products after digestion with NlaIII. The undigested PCR product is 249 bp. Upon digestion, the WT product yields 198 bp and 51 bp bands, while the *sc* product yields 151 bp, 51 bp and 47 bp bands. The *sc/+* genotype is identified by the presence of both the 198 bp and 151 bp bands. Click here for file
