# Supplementary material for: Genome-wide SNP scan of pooled DNA reveals nonsense mutation in FGF20 in the scaleless line of featherless chickens
Source: BMC Genomics. 2012 Jun 19;13:257. doi: 10.1186/1471-2164-13-257 (PMC3464622; doi:10.1186/1471-2164-13-257)
Supplement: Additional file 1 — Table S1. Results from sequencing of candidate mutations inEFHA2andSLC7A2.The polymorphisms identified in sc/sc were found to be carried by a number of WTs. The nucleotide numbers given for the EFHA2 c.144 and SLC7A2 c.1211 SNPs refer to the NCBI coding sequences; the nucleotide numbers given for the SLC7A2 polymorphisms between c.1159 and c.1164 refer to the ENSEMBL coding sequence. The letter/number codes given for each wild type sample are our identifiers for specific individuals within the diverse chicken DNA collection. Table S2 Raw data obtained from mapping of thesclocus. Names, position on chromosome 4, intensity values and absRAFdif for each SNP located within the 1.25 Mb mapped region depicted in Figure 2 are shown, together with the location of each of the 11 genes in the region (shaded). The 3 SNPs with absRAFdif values above 0.45 are highlighted in red. See Methods for explanation of data processing. [file 1471-2164-13-257-S1.doc]

| **Sample** | **Genotype** | |
| --- | --- | --- |
|  | ***EFHA2* c.1443 + 4A>C** | ***SLC7A2* c.1159_1160insA, c.1164delG** |
| Reference chicken genome | A | CTTTTG |
| *sc/sc* | C | CATTTT |
| Wild type D1, Appenzellar | C | CATTTT |
| Wild type D2, Brahma | C | CTTTTG |
| Wild type D3, Cochin | C | CATTTT/CTTTTG |
| Wild type D4, Derbyshire Redcap | C | CATTTT |
| Wild type D5, Hamburg | C | CATTTT |
| Wild type D6, Ixworth | C | CATTTT |
| Wild type D7, Lincolnshire Buff | C | CATTTT |
| Wild type D8, Marsh Daisy | C | CATTTT |
| Wild type D9, Old English Pheasant Fowl | C | CATTTT |
| Wild type D10, Scots Dumpy | C | CATTTT |
| Wild type D11, Silkie | C | CATTTT |
| Wild type D12, Light Sussex | C | CATTTT |
| Wild type G1, Araucana | C | CATTTT |
| Wild type G2, Buff Orpington | C | CATTTT |
| Wild type G3, Croad Langshan | C | CATTTT |
| Wild type G4, Dorking | C | CATTTT |
| Wild type G5, Indian Game | C | CATTTT |
| Wild type G6, Leghorn (coloured) | C | CATTTT |
| Wild type G7, Maran | C | CTTTTG |
| Wild type G8, Norfolk Grey | C | CATTTT |
|  | ***SLC7A2* c.1211G>C** | |
| Reference chicken genome | G | |
| *sc/sc* | C | |
| Wild type C12, Light Sussex | C/G | |
| Wild type B2, Brahma | G | |
| Wild type B3, Cochin | G | |
| Wild type E4, Dorking | C | |
| Wild type E5, Indian Game | C | |
| Wild type B6, Ixworth | C | |
| Wild type B7, Lincolnshire Buff | C | |
| Wild type B8, Marsh Daisy | C | |
| Wild type B9, Old English Pheasant Fowl | C | |
| Wild type B10, Scots Dumpy | C | |
| Wild type B11, Silkie | G | |
| Wild type B12, Light Sussex | C/G | |
| Wild type F1, Araucana | C | |
| Wild type F2, Buff Orpington | C | |
| Wild type F3, Croad Langshan | C/G | |
| Wild type F4, Dorking | C | |
| Wild type F5, Indian Game | C | |
| Wild type F6, Leghorn (coloured) | C | |
| Wild type F7, Maran | G | |
| Wild type F8, Norfolk Grey | C/G | |

**TABLE S1**

Results from sequencing of candidate mutations in *EFHA2* and *SLC7A2*. The polymorphisms identified in *sc/sc* were found to be carried by a number of WTs. The nucleotide numbers given for the *EFHA2* c.144 *and SLC7A2* c.1211 SNPS refer to the NCBI coding sequences; the nucleotide numbers given for the *SLC7A2* polymorphisms between c.1159 and c.1164 refer to the ENSEMBL coding sequence. The letter/number codes given for each wild type sample are our identifiers for specific individuals within the diverse chicken DNA collection.

| **Position (Mb)** | **absRAFdif** | **start/stop** | **Gene** | **SNP Name** | **RAF scsc - sc+** | **scsc X/(X+Y)** | **sc+ X/(X+Y)** | **sc+ X** | **sc+ Y** | **scsc X** | **scsc Y** |
| --- | --- | --- | --- | --- | --- | --- | --- | --- | --- | --- | --- |
| **64.773116** | **0.485687085** |  |  | **GGaluGA262579** | **0.485687085** | **0.874591058** | **0.388903973** | **4297** | **6752** | **5614** | **805** |
| 64.774096 |  | Start | MTUS1 |  |  |  |  |  |  |  |  |
| 64.815983 | 0.287760112 |  |  | Gga_rs14480992 | 0.287760112 | 0.954642192 | 0.66688208 | 17543 | 8763 | 21531 | 1023 |
| 64.842357 |  | Stop | MTUS1 |  |  |  |  |  |  |  |  |
| 64.845474 |  | Start | PDGFRL |  |  |  |  |  |  |  |  |
| 64.851050 | 0.084477662 |  |  | GGaluGA262601 | 0.084477662 | 0.708011387 | 0.623533724 | 1701 | 1027 | 1741 | 718 |
| 64.865679 |  | Stop | PDGFRL |  |  |  |  |  |  |  |  |
| 64.872326 |  | Start | SLC7A2 |  |  |  |  |  |  |  |  |
| 64.877515 | 0.290438341 |  |  | Gga_rs14481131 | 0.290438341 | 0.896443601 | 0.60600526 | 8295 | 5393 | 10864 | 1255 |
| 64.910696 |  | Stop | SLC7A2 |  |  |  |  |  |  |  |  |
| 64.915020 | 0.042136018 |  |  | Gga_rs14481174 | 0.042136018 | 0.140063273 | 0.182199291 | 565 | 2536 | 487 | 2990 |
| 64.931603 |  | Start | MTMR7 |  |  |  |  |  |  |  |  |
| 64.936174 | 0.224400185 |  |  | Gga_rs14481185 | 0.224400185 | 0.064530147 | 0.288930332 | 2169 | 5338 | 594 | 8611 |
| 64.949449 | 0.433563195 |  |  | Gga_rs14481207 | 0.433563195 | 0.934883049 | 0.501319854 | 8926 | 8879 | 12950 | 902 |
| 64.977472 |  | Stop | MTMR7 |  |  |  |  |  |  |  |  |
| 64.981226 |  | Start | VPS37A |  |  |  |  |  |  |  |  |
| 64.988633 | 0.340459191 |  |  | Gga_rs16424343 | 0.340459191 | 0.913782852 | 0.57332366 | 5532 | 4117 | 7684 | 725 |
| 64.994230 |  | Stop | VPS37A |  |  |  |  |  |  |  |  |
| 64.995708 |  | Start | CNOT7 |  |  |  |  |  |  |  |  |
| 65.015117 |  | Stop | CNOT7 |  |  |  |  |  |  |  |  |
| 65.024648 |  | Start | ZDHHC2 |  |  |  |  |  |  |  |  |
| 65.033826 | 0.010227949 |  |  | Gga_rs16424383 | 0.010227949 | 0.872598198 | 0.882826147 | 19205 | 2549 | 20527 | 2997 |
| 65.039229 | 0.140272332 |  |  | Gga_rs14481301 | 0.140272332 | 0.931806241 | 0.79153391 | 6956 | 1832 | 8868 | 649 |
| 65.052678 |  | Stop | ZDHHC2 |  |  |  |  |  |  |  |  |
| 65.061940 |  | Start | EFHA2 |  |  |  |  |  |  |  |  |
| 65.066393 | 0.179251071 |  |  | GGaluGA262668 | 0.179251071 | 0.819796215 | 0.640545145 | 2256 | 1266 | 2816 | 619 |
| 65.090539 | 0.172857588 |  |  | Gga_rs14481362 | 0.172857588 | 0.762083177 | 0.589225589 | 1925 | 1342 | 2034 | 635 |
| 65.114060 |  | Stop | EFHA2 |  |  |  |  |  |  |  |  |
| 65.114153 |  | Start | FGF20 |  |  |  |  |  |  |  |  |
| 65.117535 |  | Stop | FGF20 |  |  |  |  |  |  |  |  |
| 65.121579 | 0.394568109 |  |  | Gga_rs14481416 | 0.394568109 | 0.923381865 | 0.528813756 | 9103 | 8111 | 12811 | 1063 |
| 65.138424 | 0.230133369 |  |  | Gga_rs14481433 | 0.230133369 | 0.084462151 | 0.31459552 | 2205 | 4804 | 636 | 6894 |
| 65.148559 | 0.051344279 |  |  | Gga_rs14481471 | 0.051344279 | 0.140876142 | 0.192220421 | 593 | 2492 | 447 | 2726 |
| 65.183811 | 0.097130507 |  |  | Gga_rs16424607 | 0.097130507 | 0.107212823 | 0.20434333 | 988 | 3847 | 602 | 5013 |
| 65.198561 | 0.177078482 |  |  | Gga_rs14481546 | 0.177078482 | 0.874446903 | 0.697368421 | 7208 | 3128 | 7905 | 1135 |
| 65.227298 | 0.059399075 |  |  | Gga_rs15601457 | 0.059399075 | 0.719260065 | 0.65986099 | 1519 | 783 | 1983 | 774 |
| 65.241354 | 0.124480897 |  |  | GGaluGA262721 | 0.124480897 | 0.237126246 | 0.361607143 | 648 | 1144 | 571 | 1837 |
| 65.261835 | 0.118571575 |  |  | Gga_rs14481608 | 0.118571575 | 0.115688209 | 0.234259784 | 826 | 2700 | 469 | 3585 |
| **65.295838** | **0.455897115** |  |  | **GGaluGA262735** | **0.455897115** | **0.943680592** | **0.487783477** | **7227** | **7589** | **10707** | **639** |
| 65.300169 | 0.260637033 |  |  | Gga_rs14481637 | 0.260637033 | 0.036311035 | 0.296948068 | 4826 | 11426 | 615 | 16322 |
| 65.327854 | 0.169105719 |  |  | GGaluGA262743 | 0.169105719 | 0.19519634 | 0.364302059 | 796 | 1389 | 512 | 2111 |
| 65.362042 | 0.060568286 |  |  | Gga_rs14481661 | 0.060568286 | 0.045684311 | 0.106252597 | 2046 | 17210 | 958 | 20012 |
| 65.378477 | 0.048045641 |  |  | Gga_rs13548779 | 0.048045641 | 0.273755656 | 0.225710015 | 453 | 1554 | 484 | 1284 |
| 65.403982 | 0.195150901 |  |  | GGaluGA262754 | 0.195150901 | 0.827743493 | 0.632592593 | 1708 | 992 | 3085 | 642 |
| 65.425309 | 0.040285104 |  |  | Gga_rs14481690 | 0.040285104 | 0.074442007 | 0.114727112 | 906 | 6991 | 577 | 7174 |
| 65.446186 | 0.261478789 |  |  | Gga_rs14481713 | 0.261478789 | 0.897475998 | 0.63599721 | 16410 | 9392 | 20659 | 2360 |
| 65.466287 | 0.06294713 |  |  | Gga_rs14481727 | -0.06294713 | 0.044205084 | 0.107152214 | 1227 | 10224 | 513 | 11092 |
| 65.474608 |  | Start | TUSC3 |  |  |  |  |  |  |  |  |
| 65.493324 | 0.055140541 |  |  | Gga_rs14481770 | 0.055140541 | 0.035160023 | 0.090300564 | 1376 | 13862 | 546 | 14983 |
| 65.513802 | 0.177464743 |  |  | Gga_rs14481796 | 0.177464743 | 0.129581152 | 0.307045895 | 950 | 2144 | 495 | 3325 |
| 65.536292 | 0.377524001 |  |  | Gga_rs13548811 | 0.377524001 | 0.875455554 | 0.497931553 | 2648 | 2670 | 5525 | 786 |
| 65.547564 | 0.355032396 |  |  | Gga_rs16424901 | 0.355032396 | 0.034268524 | 0.38930092 | 5669 | 8893 | 524 | 14767 |
| 65.571874 | 0.096195672 |  |  | Gga_rs14481865 | 0.096195672 | 0.517709118 | 0.421513446 | 674 | 925 | 687 | 640 |
| 65.588064 |  | Stop | TUSC3 |  |  |  |  |  |  |  |  |
| 65.589612 | 0.062303401 |  |  | Gga_rs13548827 | 0.062303401 | 0.034799397 | 0.097102799 | 1773 | 16486 | 739 | 20497 |
| 65.611930 | 0.081872975 |  |  | GGaluGA262793 | 0.081872975 | 0.046734182 | 0.128607157 | 1337 | 9059 | 503 | 10260 |
| 65.634753 | 0.11652991 |  |  | Gga_rs14481950 | -0.11652991 | 0.251846877 | 0.368376787 | 438 | 751 | 375 | 1114 |
| 65.642988 | 0.121307604 |  |  | Gga_rs14481960 | 0.121307604 | 0.639392168 | 0.518084564 | 1017 | 946 | 1094 | 617 |
| 65.681083 | 0.02379044 |  |  | Gga_rs14731667 | -0.02379044 | 0.672886297 | 0.696676737 | 1153 | 502 | 1154 | 561 |
| 65.688592 | 0.205594652 |  |  | Gga_rs14481971 | 0.205594652 | 0.391705069 | 0.186110417 | 2235 | 9774 | 425 | 660 |
| 65.730947 | 0.222632995 |  |  | Gga_rs16425095 | 0.222632995 | 0.030860393 | 0.253493388 | 5406 | 15920 | 651 | 20444 |
| 65.739340 | 0.277991085 |  |  | Gga_rs13548858 | 0.277991085 | 0.891015978 | 0.613024893 | 4876 | 3078 | 5911 | 723 |
| 65.763361 | 0.227858412 |  |  | Gga_rs14482026 | 0.227858412 | 0.423785595 | 0.195927183 | 635 | 2606 | 506 | 688 |
| 65.790023 | 0.090996584 |  |  | GGaluGA262828 | 0.090996584 | 0.806663026 | 0.715666442 | 3184 | 1265 | 2954 | 708 |
| 65.791859 |  | Start | SGCZ |  |  |  |  |  |  |  |  |
| 65.797201 | 0.034837405 |  |  | GGaluGA262832 | 0.034837405 | 0.742943548 | 0.708106143 | 1948 | 803 | 2211 | 765 |
| 65.831538 | 0.140497649 |  |  | GGaluGA262841 | 0.140497649 | 0.186206897 | 0.326704545 | 690 | 1422 | 486 | 2124 |
| 65.857591 | 0.053697158 |  |  | Gga_rs14482073 | 0.053697158 | 0.825393308 | 0.77169615 | 3708 | 1097 | 3725 | 788 |
| 65.899104 | 0.21618862 |  |  | Gga_rs14482115 | 0.21618862 | 0.757319224 | 0.541130604 | 1388 | 1177 | 2147 | 688 |
| 65.920557 | 0.202160745 |  |  | Gga_rs16425301 | 0.202160745 | 0.122059166 | 0.324219911 | 1091 | 2274 | 524 | 3769 |
| 65.926490 | 0.245890183 |  |  | Gga_rs14482124 | 0.245890183 | 0.036355937 | 0.28224612 | 4383 | 11146 | 601 | 15930 |
| 65.970742 | 0.246704796 |  |  | Gga_rs14482147 | 0.246704796 | 0.790459206 | 0.543754411 | 1541 | 1293 | 1773 | 470 |
| 65.980227 | 0.272610345 |  |  | GGaluGA262866 | 0.272610345 | 0.081282009 | 0.353892354 | 1532 | 2797 | 492 | 5561 |
| 66.009865 | 0.265354543 |  |  | GGaluGA262868 | 0.265354543 | 0.871266968 | 0.605912425 | 2726 | 1773 | 3851 | 569 |
| **66.022973** | **0.47535652** |  |  | **GGaluGA262871** | **0.47535652** | **0.797841727** | **0.322485207** | **1635** | **3435** | **2218** | **562** |
| 66.055249 | 0.213660783 |  |  | Gga_rs14482197 | 0.213660783 | 0.8 | 0.586339217 | 1528 | 1078 | 2512 | 628 |
| 66.075062 |  | Stop | SGCZ |  |  |  |  |  |  |  |  |
| 66.078516 | 0.116056733 |  |  | Gga_rs14482208 | 0.116056733 | 0.050362286 | 0.166419019 | 1792 | 8976 | 563 | 10616 |

**TABLE S2**

Raw data obtained from mapping of the *sc* locus. Names, position on chromosome 4, intensity values and absRAFdif for each SNP located within the 1.25 Mb mapped region depicted in Figure 2 are shown, together with the location of each of the 11 genes in the region (shaded). The 3 SNPs with absRAFdif values above 0.45 are highlighted in red. See Methods for explanation of data processing.

**SUPPLEMENTARY MATERIAL**

**Oligonucleotides used for amplification and sequencing of *sc* candidate genes**

***FGF20 (*XM_426335.3)**

**Genomic DNA oligos:**

**Oligo name Sequence (5’–3’)**

FGF20_1F GACCCTTCAGGACACAGCAT

FGF20_1R CCCAGACACACATGAAAGGA

FGF20_2F AAGTACGAGGGAAAATTTGCAG

FGF20_2R GTGGGCATGTGGGGCTAC

FGF20_3F GGAGCGGGTTTACAATTTCA

FGF20_3R TAAGCACCCCAGAAACGAAC

FGF20_4R GCCATTCCAGCAGATTGTTT

FGF20_5F GGAATGCAGCATCTGAACAA

FGF20_5R CTGCAGTTTGGCAAGTGTGT

FGF20_6F CCACTCTATCAACAGCAAAATCT

FGF20_6R TTTCCCCCACCCTTAATTT

FGF20_7F TGATGTTTGAGCAGGCAACT

FGF20_7R TGCACTGCACAAAGGAAAAC

FGF20_8F AGCACTATGCAAGCACTAAAAA

FGF20_8R CACATATTCAATTTTGGATTTAAACG

FGF20_9F TGGTAGACTCCCCATGACAG

FGF20_9R TACTGGCTGCCACACAGTTC

FGF20_10F ATACTGGCACAGGGGATTTG

FGF20_10R AATTCCCTTTTTCAAATGTGC

FGF20_11F CAGATAAGGTTATATTTTGTGCTCAT

FGF20_11R CTGTAGAGGTGCCAGCCATC

***SGCZ (*XM_429984.3)**

**cDNA oligos:**

SGCZ_120112_2 CCATCAGACTCTGGGCAAAT

SGCZ_030112_F ATCCTTGGTGATGGAAGCAC

SGCZ_030112_R ATGGAAGAGCTCGGTGAAGA

SGCZ_170212_5 ACAGTGGATGGAATGGGAAA

**gDNA oligos:**

SGCZ_gDNA_010212_F TTAATGCATCCCTTCTGTGC

SGCZ_gDNA_010212_R TAAGCACCTCTTTCGCCATC

SGCZ_170212_Ex1F TGCCCATAGAAACTGATTGC

SGCZ_170212_Ex1R GGGCAAAGTTGCACTTACATC

SGCZ_170212_Ex2F TGGGACTTTTGACTTCACCA

SGCZ_170212_Ex2R TGACATGAAGGAAGATCTCAGTG

***TUSC3 (*XM_420692.3)**

**cDNA oligos:**

TUSC3_030112_F GCTCCGACTTTCATGCATTT

TUSC3_030112_R GAGGTCCACGGATGTGATTC

TUSC3_120112_1 GGAGGCCAGCGGCTAACG

TUSC3_120112_2 CAGATGAGTAGCGCCATGAA

TUSC3_120112_3 ACAGTGGTGGGCAGATAAGC

***EFHA2 (*XM_420691.3)**

**Based on EST evidence and the ENSEMBL gene model, the *EFHA2* coding sequence was deduced to start at NCBI c.766.**

**cDNA oligos:**

EFHA2_030112_R TTGACCTATGGAACGACTTGC

EFHA2_120112_4 GACACCACCAGTTTGGAAGG

EFHA2_120112_6 AAAACATGCGCTGCAGGA

EFHA2_120112_7 CGAAAAAGACTTGTGCCAAT

**gDNA oligos:**

EFHA2_170212_Ex1F GTTTCTCGCGAGGTTTTCTC

EFHA2_170212_Ex1R CAGCCCTCAGCCGACTCC

EFHA2_170212_Ex2F CAGCAGTATTTGGATGGATTTG

EFHA2_170212_Ex2R CACCAATCGCAAACAACAGT

EFHA2_170212_Ex3F GCTTCAATCTGATGAATACAGTAGG

EFHA2_170212_Ex3R CTGTGACTGCAAGTTCACATCA

EFHA2_170212_Ex4F TGCATTTCCTTTCCTTTGCT

EFHA2_170212_Ex4R GCACAGGTAGGCTTCCAGAC

EFHA2_170212_Ex5F TTTAGTCAGCTAATGCAAACAAAAA

EFHA2_170212_Ex5R TGGGATAGGCAGAAGAAAACA

EFHA2_170212_Ex6F GCTGTGTAATTTCTTAGGGCTTC

EFHA2_170212_Ex6R AAAATAAAGGCCACGTGCTG

EFHA2_170212_Ex7F TGTTACCTGTGGTGAATGACTG

EFHA2_170212_Ex7R AAGCACTTTCCCCACAACTG

EFHA2_170212_Ex8F CCATCTTCTATCCACTGTCACC

EFHA2_170212_Ex8R AGCACATGCAACCTTTCACA

EFHA2_170212_Ex9F TTGAGCCATGTTTCTGAGGA

EFHA2_170212_Ex9R TGTATAATGAAGAAAAGAAACAAAACA

EFHA2_170212_Ex10F TGCTATGAGCTTCAAACACAAAA

EFHA2_170212_Ex10R ATGCAGCACAAAGGGAAAAT

EFHA2_170212_Ex11F CAAGGCTTTTAATGGACATGC

EFHA2_170212_Ex11R AACATCCTTGGCTCCCTACA

EFHA2_170212_Ex12F GCAAGGTTTGTTTATTCCCTTG

EFHA2_170212_Ex12R GGAATGAAAAGCCAATTTCC

EFHA2_gDNA_010212_F ACCCCTTCCCCGTTTCTC

EFHA2_gDNA_010212_R CACACCTACCGTCTCCTTGG

***ZDHHC2 (*XM_420689.3)**

**NCBI and ENSEMBL predict different first exons for *ZDHHC2*. Based on EST evidence, the ENSEMBL prediction was judged to be correct. The ENSEMBL exon 1 was sequenced along with the NCBI coding sequence beginning at c.46.**

**cDNA oligos:**

ZDHHC2_120112_2 TCATTGTCCTTGGGTGAACA

ZDHHC2_120112_4 CATCCACGTAGGAGCAGACA

**gDNA oligos:**

ZDHHC2_050312_EEx1_F GGAGGAGCGCGGAAGGAG

ZDHHC2_050312_EEx1_R AAGGGGAGCGCTAACTGTG

ZDHHC2_gDNA_010212_F2 ACTGCAGCTATACGGCCAAC

ZDHHC2_gDNA_010212_R2 CTCACATGGATCAGCAATGG

ZDHHC2_170212_Ex2F CAACTGCCCTGTAAAAGTGTGA

ZDHHC2_170212_Ex2R TGCATCTCAATTTATTGGAAAA

ZDHHC2_170212_Ex3F CCCCATGCAGTCTGTATTTGT

ZDHHC2_170212_Ex3R TCGTGAAAAACCACATGAGA

ZDHHC2_170212_Ex4F CCTTGTACATGCTGCTAATCCA

ZDHHC2_170212_Ex4R TTCTTCTGTACCATCCAAATCC

ZDHHC2_170212_Ex5F GCTTCAGGGTTTGCAAGTTC

ZDHHC2_170212_Ex5R TGCATGTAAAATTCTGCATCAAG

ZDHHC2_170212_Ex6F TGCATGTGTAATGCTGATGTG

ZDHHC2_170212_Ex6R TGGGCAGCCTGGTCTAGTAT

ZDHHC2_170212_Ex7F TTCAGTAGCTTGAGGCACCA

ZDHHC2_170212_Ex7R CGTGCAATTTGGTTTTTGAC

***CNOT7 (*NM_001006454.1)**

**cDNA oligos:**

CNOT7_030112_R GCTTTCCCCTTCTCTCTGCCACAC

CNOT7_120112_1 GGCGGGGTGAGGTAAAAA

CNOT7_120112_2 CTGCGATTGTTTTTCCCTGT

CNOT7_120112_3 GCCTGATGCTGTGGTCCTAT

***VPS37A (*XM_420687.3)**

**cDNA oligos:**

VSP37A_120112_1 GAGGTTTCGCCGGTGTCC

VSP37A_120112_2 TTTACAGTGAACGGCAGTCG

VSP37A_120112_3 TGCTTCTGAATCGGTTTCTTC

VSP37A_120112_4 GTAAATCCGTTCTGGCCAAC

VSP37A_120112_5 TCCCAGCTCTGTATACAATCTCTTC

***MTMR7 (*XM_420686.3)**

**cDNA oligos:**

MTMR7_120112_1 CATGGCTGCCCGCCTGAC

MTMR7_120112_3 AGGCTGGCTGCTCCTACATATT

MTMR7_120112_4 GAAAAAGACTGGGTTTCCTTTG

MTMR7_120112_6 CTGGAAGAAGAGCTGGCAGT

MTMR7_120112_7 CTGGAAGCAGAAAGCGCTAACTC

MTMR7_120112_8 CCACAGACAATTCCCATCCT

**gDNA oligos:**

MTMR7_170212_ex5F TAATAAATGCTTCGGGTTGC

MTMR7_170212_ex5R GTGTCAGCCATTTGGGCTAT

MTMR7_170212_ex6F CAGTGCCAGCGTAGTTGTGT

MTMR7_170212_ex6R CAAATCCATGAGCTAGCAGGT

MTMR7_170212_ex7F CAGCCCTCTTTCTCAAATGC

MTMR7_170212_ex7R CAAAATGTCCATGCAACAGC

MTMR7_170212_ex8F TTTCCCAGTTCCCAAATGAA

MTMR7_170212_ex8R CCAGCTGAAAAGGCACACTT

MTMR7_170212_ex9F ACCATTCTTCTGCCTGCTTC

MTMR7_170212_ex9R TGCTCAAGGCATTTTGTTGA

***SLC7A2 (*NM_001199102.1)**

**NCBI and ENSEMBL predict different exon structures for *SLC7A2*. All exons from both prediction methods were sequenced and no mutations were found.**

**cDNA oligos:**

SLC7A2_120112_1 TTTCAGAGCTCTCCATCCAC

SLC7A2_120112_2 CACCAGCACCAAGGGTACTT

SLC7A2_120112_3 TCTGGTTTTGTGAAAGGAGATG

SLC7A2_120112_4 ATGGCATAAAGCCACCACTC

SLC7A2_120112_5 CACTTGTGGCAACCTGTGTC

SLC7A2_120112_7 AGAGGCACAACGTTGGTAGG

SLC7A2_170212_8 GAGGGATCCCACTGCTACAA

**gDNA oligos:**

SLC7A2_170212_ex5F AAACACATTTCTTTGTCTGTGATGA

SLC7A2_170212_ex5R GCATGAAGTGAGCCTTCAAA

SLC7A2_170212_ex6EF CAGAAAACCAAGAGCAGCAA

SLC7A2_170212_ex6eR AACAAGCAGCCCTTTTTCAA

SLC7A2_170212_ex7F GGTTCAGGAATGTTGGCTATG

SLC7A2_170212_ex7R CCATTGTAGCGTGACTCCTG

***PDGFRL (*XM_001231867.2)**

**cDNA oligos:**

PDGRFL_120112_2 AAGAGGAACTTTTTGTACCTACTCC

PDGRFL_120112_3 CTGCTGGAAATTCCCTGTGT

PDGRFL_120112_4 GGCCTGGCATTCTAGTGAAAAT

PDGFRL_170212_5 GCTTCAGCTGTGCAATGGTA

PDGFRL_170212_6 AATCTGAGGTGCTCCCTGTG

PDGFRL_cDNA_010212 ATGCGGCTCTGGGTGCTG

**gDNA oligos:**

PDGFRL_gDNA_010212 CAGAGAGCGGAGGAGCTG

PDGRFL_120112_1 CTCCTCCCCGCCCCGAAG

***MTUS1 (*XM_420684.3)**

**cDNA oligos:**

MTUS1_120112_1 TCCTGCACTCAGTTCCTGTG

MTUS1_120112_2 CTTTCGGCGAAGATCCTAGA

MTUS1_120112_3 GCACTCAGCAAGGAAAGTCA

MTUS1_120112_4 CTGTGGAGGTTTGGGTCTGT

MTUS1_120112_5 TCCAGGAGCAGGTGGACAA

MTUS1_120112_6 TTCCTGAGCTCCGAAAAAGA

MTUS1_120112_7 GCTCTTGCTCCTGCAATTAAAGTTATGA
