# Supplementary figures and images for: Genome-wide SNP scan of pooled DNA reveals nonsense mutation in FGF20 in the scaleless line of featherless chickens
Source: BMC Genomics. 2012 Jun 19;13:257. doi: 10.1186/1471-2164-13-257 (PMC3464622; doi:10.1186/1471-2164-13-257)

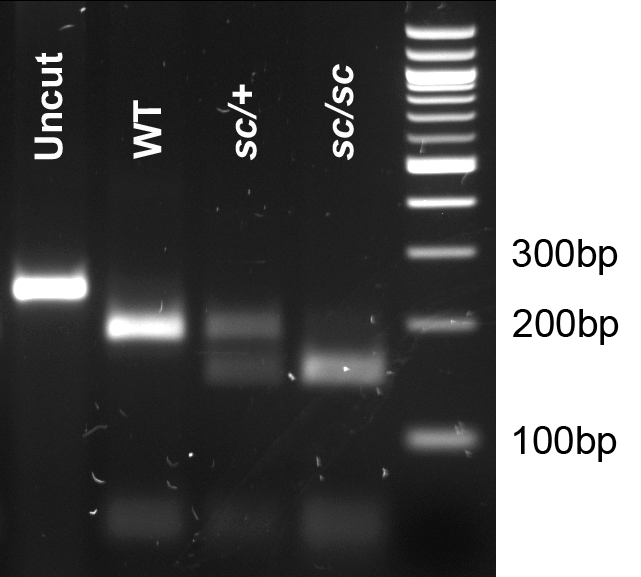

Supplement: Additional file 2 — Figure S1.Genotyping assay to discriminateFGF20 c.535A andFGF20 c.535T alleles. Agarose gel analysis of undigested dCAPS PCR product, and WT, sc/+, and sc/sc products after digestion with NlaIII. The undigested PCR product is 249 bp. Upon digestion, the WT product yields 198 bp and 51 bp bands, while the sc product yields 151 bp, 51 bp and 47 bp bands. The sc/+ genotype is identified by the presence of both the 198 bp and 151 bp bands. [file 1471-2164-13-257-S2.tiff]
